# Study for the validation of evaluation indicators of electronic health records in immunization: Delphi technique

**DOI:** 10.1590/0034-7167-2023-0112

**Published:** 2024-11-22

**Authors:** Cleide Henriqueta Praxedes Fernandes, Maria Aparecida Araújo Figueiredo

**Affiliations:** I Escola de Saúde Pública da Bahia Profº Jorge Novis. Salvador, Bahia, Brazil; II Universidade do Estado da Bahia. Salvador, Bahia, Brazil

**Keywords:** Validation Study, Immunization Programs, Electronic Health Records, Information Technology, Delphi Techniques, Estudio de Validación, Programas de Inmunización: Registros Electrónicos de Salud: Tecnología de la Información, Técnica Delfos

## Abstract

**Objective:**

To develop and validate indicators for the evaluation of computerized systems in vaccination rooms.

**Methods:**

Methodological study. From the construction of a logical model for managing information produced in computerized systems in vaccination rooms, an evaluation indicator matrix was developed, and its contents were validated by specialists using the Delphi method. The degree of relevance and clarity were judged, using the following parameters: agreement percentage ≥ 90%; content validity index > 0.78. Internal consistency was tested using Cronbach’s alpha coefficient of 0.93.

**Results:**

Of the 55 proposed indicators, 48 were validated and composed the final matrix, with 13 in the structure dimension, 29 in the process dimension, and six in the outcome dimension.

**Conclusion:**

The set of indicators shows validity and high reliability, and can be used to evaluate computerized systems in vaccination rooms throughout the country, as it adhered to the recommendations of the National Immunization Program.

## RESUMEN

**Objetivo:**

Desarrollar y validar indicadores para la evaluación de un sistema informatizado en salas de vacunación.

**Métodos:**

Estudio metodológico. A partir de la construcción de un modelo lógico de gestión de la información producida en un sistema informatizado en salas de vacunación, se elaboró una matriz de indicadores de evaluación, cuyos contenidos fueron validados por expertos utilizando el método Delphi. Se juzgó el grado de relevancia y claridad, teniendo como parámetro: porcentaje de concordancia ≥ 90%; índice de validez de contenido > 0,78. La consistencia interna fue probada con el coeficiente de Cronbach de 0,93.

**Resultados:**

De los 55 indicadores propuestos, 48 fueron validados y compusieron la matriz final, siendo 13 en la dimensión estructura, 29 en la dimensión proceso y seis en la dimensión resultado.

**Consideraciones finales:**

El conjunto de indicadores presenta validez y alta fiabilidad, pudiendo ser utilizado para evaluar el sistema informatizado en salas de vacunación en cualquier parte del país, ya que respetó las recomendaciones del Programa Nacional de Inmunizaciones.

## INTRODUCTION

In Brazil, the technological development of information systems has been marked by both advances and setbacks. Despite efforts to improve existing systems for better quality in data recording, collection, and processing, challenges remain regarding the operationalization of these systems and the fulfillment of the role of information produced in the health context ^(1)^. Nevertheless, given the diverse realities across Brazilian states, the National Policy on Health Information and Informatics has guided the information and communication technologies used in the Unified Health System (SUS), promoting the standardization of data collection and processing methods and reducing the heterogeneity of various existing information systems ^(2)^.

Within the National Immunization Program (PNI), the first efforts to systematize immunization information in epidemiological surveillance began in 1975 with the implementation of the Applied Dose Registration System ^(3)^. For many years, records remained on paper, leading to limitations in real-time data availability, potential deterioration of data integrity, compromised information quality, difficulty in data retrieval, susceptibility to information biases, and obstacles to prompt individual patient data research ^(4)^.

With technological advancements, there has been a global interest in replacing physical records with electronic records at all levels of healthcare, including immunization services ^(5)^. Following this global trend, Brazil developed the Nominal Information System of the National Immunization Program (SIPNI in Portuguese), with the main objective of individually recording the vaccination data of every Brazilian.

The introduction of a computerized system in the immunization field enabled the swift production of official vaccination records for both services and management, generating detailed information ranging from the movement of immunobiologicals in each vaccination room, whether in the public or private network, to monitoring each citizen’s vaccination history ^(6,7)^. The system allows for the individual monitoring of vaccination status, identifying individuals behind on vaccinations, automatically sending alerts about expired doses, and reminders for upcoming doses to be administered ^(8)^.

In practice, the immunization program faces significant challenges, such as the need to identify unvaccinated or under-vaccinated populations. In this context, the implementation of a computerized immunization system has provided the benefit of identifying this population to ensure that everyone is adequately immunized, promoting the maintenance of high vaccination coverage levels and reducing cases of vaccine-preventable diseases ^(9,10)^.

Thus, there is a global effort to implement electronic immunization records. Some countries are in advanced stages of utilizing computerized immunization systems, with some being individualized systems and others functioning as integrated components of computerized health management systems ^(11)^.

In Brazil, aiming to improve the quality of immunization data and enable the feeding of the information system in all vaccination rooms across Brazilian municipalities, ensuring timely data entry and regular data transmission to the national database, Ordinance No. 2,499, issued in September 2019, mandated the use of a single system for immunization data registration, the e-SUS Primary Care (e-SUS AB in Portuguese) ^(12)^. This integration brought significant changes in the automatic availability of health user information and also the advantage of organizing citizen data in one place through the electronic health record, allowing Primary Care professionals to monitor the vaccination history of health service users. Furthermore, the integration of SIPNI with e-SUS AB added and/or optimized several functionalities aimed at improving records and monitoring individuals’ vaccination status, such as the automatic scheduling of subsequent doses ^(13)^.

However, as SIPNI is a technological innovation in the management of immunization actions, it is necessary to adopt initiatives for awareness, training, supervision, and evaluation of the full utilization of the resource. Additionally, it is essential to recognize that improving system operational practices will support the implementation of more effective surveillance actions ^(14)^.

In this dynamic context of the implementation and operation of information systems, having evaluation parameters is crucial. According to Contandriopoulos and collaborators ^(15)^, during the development of health systems and services, it is important to conduct evaluations that seek to make value judgments about the proper operationalization of the intervention, establishing a comparison between planned and effectively implemented characteristics.

From this perspective, the main justification for conducting this study was the need to construct and validate indicators capable of evaluating computerized systems in vaccination rooms, based on PNI recommendations for best practices in executing vaccination actions, the performance of vaccination rooms through monitoring daily and monthly activities, and the management of immunobiologicals. The construction and validation of these indicators will enable the evaluation of computerized vaccination room systems, which, having used PNI parameters, can be applied in municipalities throughout the country.

## OBJECTIVE

To develop and validate indicators for evaluating computerized systems in vaccination rooms.

## METHODS

### Ethical Aspects

As this research is in the health field, it complied with the requirements of Resolutions No. 466/2012 and 510/2016 of the National Health Council, which governs research involving human subjects. The research was submitted to the Research Ethics Committee of the University of the State of Bahia and was approved on May 27, 2020.

### Study Design, Period, and Location

Studies aimed at validating and evaluating tools or research methods are considered methodological studies ^(16,17)^. Thus, this is a methodological study structured for the development and content validation of indicators for evaluating computerized systems in vaccination rooms. The research was conducted from December 2020 to January 2021 in the state of Bahia, Brazil.

### Population and Selection Criteria

For the selection of expert judges to validate the indicators constructed for evaluating computerized systems in vaccination rooms, based on PNI recommendations, theoretical knowledge and practical experience were primarily considered. The selection criteria were: i) having a background in health or related areas; ii) working in the management of immunization services at any government level (municipal, state, or federal) with at least two years of experience in the area. Based on these criteria, potential experts were intentionally selected and contacted via email and/or WhatsApp® messages through an electronically sent invitation letter explaining the study. Those who did not respond to the invitation letter and questionnaire within the established return period (15 days after the first contact) were excluded.

For those who agreed to participate, the informed consent form (ICF) and the questionnaire were sent. The material directed to these judges was structured in two parts: the first with questions for participant characterization and the second with the questionnaire and filling instructions. The group of expert judges consisted of 12 nurses and one nursing technician, including two master’s degree holders, 10 with specializations, and one with a secondary education level. All were female, with the majority self-identifying as brown (69.2%), aged between 31 to 40 years (76.9%), and having worked in immunization services or the PNI for 5 to 15 years (53.8%). Regarding the level of work, 84.6% worked in municipal management, with five in immunization services at health units, two in sanitary districts, and four in municipal coordination. The remaining two were state immunization coordinators.

### Study Protocol

The preliminary phase of constructing the indicators comprised an integrative literature review, accompanied by a bibliographic survey and document analysis of PNI recommendations for computerized systems in vaccination rooms. The integrative review aimed to identify the approaches adopted in evaluating computerized immunization systems in various countries and the lessons that could be drawn from the main results and conclusions of these evaluations to inform best practices and improve these systems.

The search descriptors “Immunization Programs,” “Health Evaluation,” “Immunization,” and “Health Information System” were combined in five sequences using the boolean operator “and.” The review included freely available full-text digital productions in English, Spanish, and Portuguese that addressed the evaluation of computerized immunization systems in any geographic context from January 2015 to August 2020. Productions that did not fully or partially respond to the guiding question of the review or dealt with the evaluation of computerized health systems not related to immunization were excluded. In addition to scientific articles, non-conventional documents forming the gray literature (theses, dissertations, symposium and congress proceedings, manuals, and official publications - informational notes, ordinances, and decrees) were also included.

The collection period took place from April 2019 to August 2020, conducted by a single reviewer. The selected documents were evaluated for their relevance to the review topic, and the data were extracted and organized into a descriptive spreadsheet, including information such as title, author, year, type of publication, journal, place of publication, objectives, methodology, results, conclusions, and main findings. This analysis allowed a deeper understanding of the identified evaluative studies.

The integrative review process involved the identification, screening, eligibility, and inclusion of relevant studies. Initially, 533 records were identified through the Virtual Health Library database search, and 18 additional records were found through Google Scholar. After removing duplicates, 497 records were screened by reading titles and abstracts, resulting in the exclusion of 428 records. Next, 69 full-text articles were assessed for eligibility, with 16 being excluded based on predefined criteria.

In the end, a total of 53 documents related to the Computerized Immunization System were analyzed, including 34 articles, two doctoral theses, five master’s dissertations, three symposium proceedings, one editorial, two books, one manual, one Presidential Decree, one Ministerial Informational Note, and three Ministerial Ordinances. From this document analysis, elements necessary for the functioning of computerized systems in vaccination rooms were identified. With these elements, a logical model was created as the basis for constructing the indicators.

**Figure 1:**
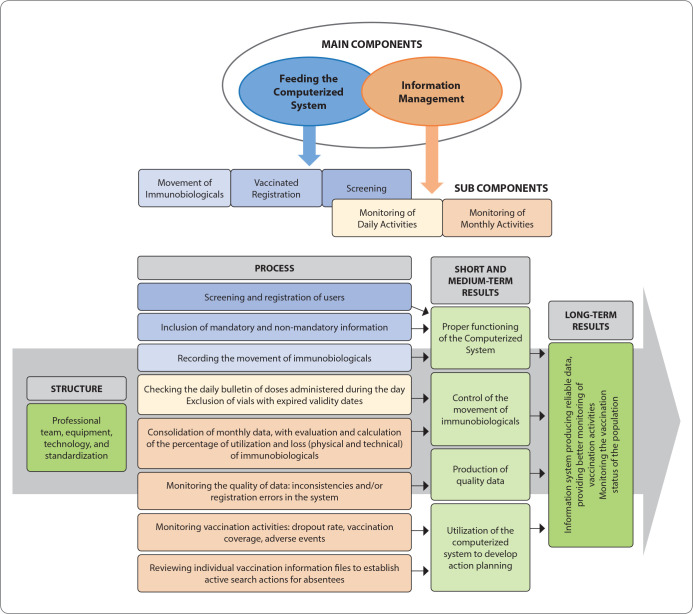
Logical Model

The logical model of a program helps in its description as it outlines how the program will be implemented and what the expected results are ^(18)^. In the logical model constructed here (Figure 1), the Donabedian dimensions of evaluation were observed ^(19)^, which consider: structure as the dimension related to the necessary human, material, and organizational resources for the operation and implementation of the system; process as the dimension that seeks to distinguish the activities carried out in the operation; and outcomes as the dimension describing the intended achievements. Subsequently, two main components were established: i) feeding the computerized system; and ii) information management. The subcomponents of feeding the computerized system were three: i) screening, ii) vaccinated registration, and iii) movement of immunobiologicals. For information management, the subcomponents were: i) monitoring daily activities and ii) monitoring monthly activities. Based on this logical model, the indicator matrix was defined.

Indicators are methodological resources, either quantitative or qualitative, that allow the analysis of variables or sets of variables constituting the observed object, important for addressing a problem. However, it is necessary to consider their relevance and utility in their definition ^(20)^. From this perspective, for each dimension (structure; process; outcome) and their respective subdimensions, at least one indicator was created, with their respective evaluation parameters and scoring criteria, thus forming the so-called “indicator matrix.” This matrix was composed of 55 indicators, with 14 proposed for the “structure” dimension, 35 for the “process” dimension, and six for the “outcomes” dimension.

To validate the content and obtain consensus on the indicator matrix, the Delphi method was used defined as a structured communication process that seeks to obtain consensus of opinions from a group of experts on a particular subject through questionnaires interspersed with controlled opinion feedback. The method presupposes that the collective opinion of experts, when properly organized, surpasses individual judgment ^(21,22)^. Here, the Modified e-Delphi was followed, which consists of a process similar to the classic Delphi, administered via email or online through web surveys ^(23)^. Thus, the matrix was sent to the experts via a Google Forms® electronic spreadsheet.

At the beginning of the Delphi method application, it is important to structure the instruction note for the participants, providing clarifications about the execution of the investigation, the structure of the questionnaires, the criteria, and the evaluation method^(23)^, understanding that consensus is reached when the opinions of the judges converge^(24)^.

Thus, the judges were asked to evaluate each indicator according to its relevance and clarity, using the following scale: i) relevance - understood as the degree of importance (high for essential indicators without which it is not possible to offer the planned service as they cover fundamental requirements for its operation; medium for necessary indicators that should be present and point to the adoption of best practices in the organization and operation of the service, but are not essential; low for indicators that may be present in the organization and operation of the service but require significant revision to be relevant; and null for irrelevant indicators); ii) clarity - assessing whether the indicator is written clearly, simply, easily, and naturally, with “yes” if the question is considered clearly written, and “no” if it is considered unclear.

During the first round, out of the 26 professionals contacted, 14 responded to the invitation letter. Of these, one did not respond to the questionnaire, resulting in a first-round group composed of 13 professional judges, with an abstention rate of 7.14%. For the second round, there was an abstention rate of 53.8%, with only six of the 13 specialists responding.

### Analysis of results and statistics

The data were stored in an Excel® spreadsheet. To validate the content of an instrument, different methods exist to quantify the degree of agreement among expert judges ^(25)^. For this study, the percentage of agreement among the judges and the Content Validity Index (CVI) were used. Following the recommendations of Polit and Beck, indicators with an agreement percentage below 90% (for clarity) or a CVI below 0.78 (for relevance) were reformulated or excluded from the instrument ^(16)^. The following formulas were used:


$$\text{Percentage of Agreement}=\frac{\text{Number of participants who agree (yes) on clarity× 100}}{\text{Total number of particints}}$$

$$\text{CVI}=\frac{\text{Number of responses considering relevance as high or medium}}{\text{Total number of responses (high, medium, low, or null relevance)}}$$

To estimate the internal consistency of the questions and answers, Cronbach’s coefficient was calculated using the open-source statistical package R, with desirable values ranging between 0.70 and 1.0^(26)^.

## RESULTS

Of the 26 professionals contacted, 14 responded to the invitation letter. Of these, one did not respond to the questionnaire, resulting in a first-round group of 13 experts, with a 7.1% abstention rate. For the second round, there was an abstention rate of 53.8%, as only six of the 13 participants from the first round responded.

In the first round, 80% of the 55 indicators achieved an agreement percentage and CVI above the pre-established values, thus being considered as having valid content. For the structure dimension, which addressed structural, human, material, and organizational resources, 12 out of the 14 indicators were validated in the first round, while two did not achieve consensus regarding their clarity and/or relevance. These were: “to carry out or request the inclusion of new professionals in the computerized system” and “absence of a registered professional in the computerized system who is no longer part of the staff.” The non-validated indicators were reformulated and presented to the judges in the second round, with one being excluded at this stage (Chart 1).

**Chart 1:** Indicators of the computerized system in vaccination rooms related to the structure dimension not validated in the first round of the Delphi method and their status after the second round

Indicator presented in the 1st round	1st Round Delphi Result		Reformulated Indicator presented in the 2nd round	Final Result
	CVI	% Agreement		
Absence of a registered professional in the SI who is no longer part of the staff.	0.77	84.6%	Professionals who no longer belong to the vaccination room staff are excluded from the SI.	Indicator excluded.
Carry out or request the inclusion of new professionals in the SI.	0.92	84.6%	All professionals working in the vaccination room are registered in the SI.	Indicator validated. (CVI = 1 and % Agreement = 100%)

*Notes: SI - Information System CVI - Content Validity Index*

**Figure 2:**
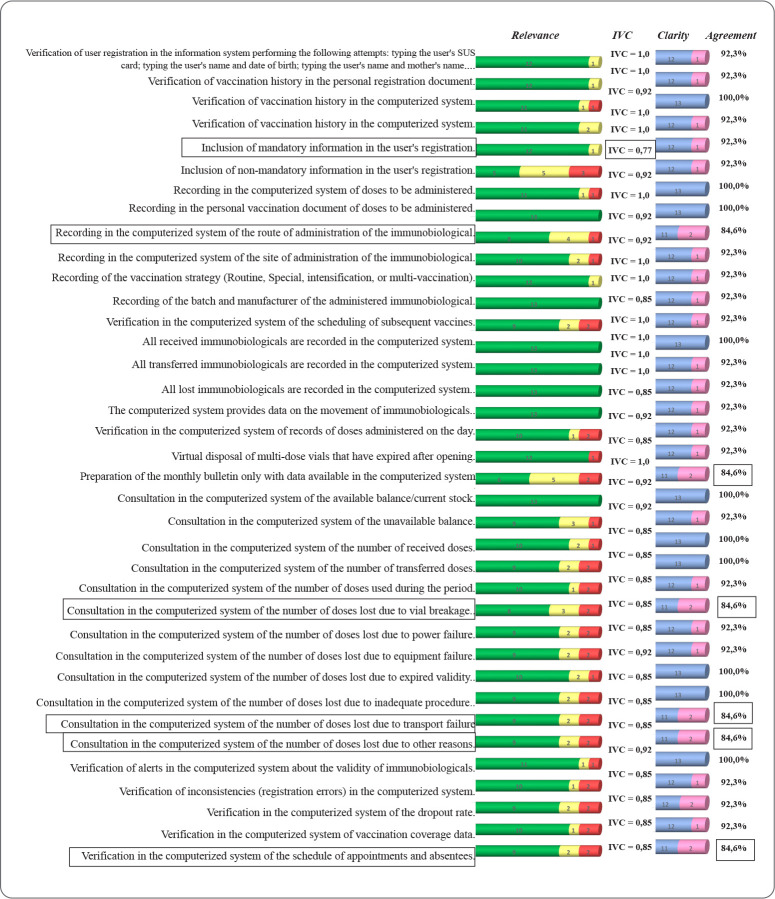
Agreement among experts for the implementation indicators of the computerized system in vaccination rooms related to the PROCESS dimension in the first round of the Delphi method, according to CVI and % Agreement

When analyzing the 35 indicators related to the process dimension, which addressed the activities carried out in the operation of the computerized system, 29 were validated in the first round, one did not achieve a CVI > 0.78, and five had an agreement below 90% (Figure 2). These were then moved to the second round. Of these, five were validated, and one was excluded at this stage (Chart 2).

**Chart 2:** Indicators of the computerized system in vaccination rooms related to the process dimension not validated in the first round of the Delphi method and their status after the second round

Indicator presented in the 1st round	1st Round Delphi Result		Reformulated Indicator presented in the 2nd round	Final Result
	CVI	% Agreement		
Inclusion of non-mandatory information in the user registration.	0.77	92.3%	Reintroduced in full	Indicator excluded.
Recording in the SI of the route of administration of the immunobiological.	0.92	84.6%	Includes in the SI data on the route of vaccine administration.	Indicator validated. (CVI = 1 and % Agreement = 100%)
Checking in the SI the number of doses lost due to vial breakage.	0.85	84.6%	Displays in the SI the record of doses lost due to vial breakage.	Indicator validated. (CVI = 0.83 and % Agreement = 100%)
Checking in the SI the number of doses lost due to transport failure.	0.85	84.6%	Displays in the SI the record of doses lost due to transport failure.	Indicator validated. (CVI = 0.83 and % Agreement = 100%)
Checking in the SI the number of doses lost due to technical losses or other reasons.	0.85	84.6%	Displays in the SI the record of doses lost due to technical losses or other reasons.	Indicator validated. (CVI = 0.83 and % Agreement = 100%)
Checking in the computerized system the schedule of appointments and absentees.	0.85	84.6%	The list of absent patients and the scheduling of subsequent doses are displayed in the SI.	Indicator validated. (CVI = 1 and % Agreement = 100%)

*Notes: SI - Information System CVI - Content Validity Index*

Of the six indicators that comprised the outcome dimension, which addresses the functionality of management reports that reflect the objectives of the computerized system, three indicators were validated in the first round. These were: “produces the monthly bulletin only with data available in the computerized system,” “reviews individual information files of vaccinated individuals recorded in the computerized system for individuals followed by the unit,” and “issues a report of vaccinated individuals by vaccine.” The other three were reformulated and validated in the second round (Chart 3).

**Chart 3:** Indicators of the computerized system in vaccination rooms related to the outcome dimension not validated in the first round of the Delphi method and their status after the second round

Indicator presented in the 1st round	1st Round Delphi Result |	Reformulated Indicator presented in the 2nd round	Final Result
Prepares the monthly bulletin only with data available in the SI.	ICV = 0,85 Concordância = 84,6%	The Monthly Bulletin is prepared exclusively with SI data.	Indicator validated. CVI = 0.83 and % Agreement = 100%
Reviews individual information files of vaccinated individuals registered in the SI and monitored by the unit.	ICV = 0,85 Concordância = 84,6%	Reviews information files of vaccinated individuals registered in the SI and monitored by the unit.	Indicator validated. CVI = 1 and % Agreement = 100%
Issues a report of vaccinated individuals by vaccine.	ICV = 0,85 Concordância = 84,6%	Displays in the SI the list of vaccinated individuals by each immunobiological.	Indicator validated. CVI = 1 and % Agreement = 100%

*Notes: SI - Information System CVI - Content Validity Index*

The final matrix consisted of 48 indicators: 13 for the STRUCTURE dimension, 29 for the PROCESS dimension, and six for the OUTCOME dimension. Cronbach’s alpha coefficient (data not shown) reached 0.93 (95% CI), revealing a significantly high value. This finding suggests robust internal consistency for the questionnaire, indicating excellent internal reliability regarding the clarity and relevance of the indicators.

## DISCUSSION

In the context of publications on evaluations of computerized systems in vaccination rooms, a study highlighted the experience of the World Health Organization (WHO), which in 2001 developed a self-assessment tool by adapting the Data Quality Audit methodology for use in national programs. The aim was to support countries in improving data collection and usage to diagnose problems at all levels of management (federal, state, municipal, or local). This methodology was refined in 2014 in partnership with the United States Centers for Disease Control and Prevention (CDC)^(8,27)^. Similarly, another study reported the experience of the Pan American Health Organization (PAHO) in developing a methodology to evaluate electronic vaccination records in lowand middle-income countries in Latin America and the Caribbean. This initiative emerged from discussions on what would be considered “ideal” for a computerized immunization system, considering the following evaluated dimensions: i) system purpose; ii) legal context and regulation; iii) software functionality; iv) maintenance; v) sustainability; vi) human resources; and vii) modules included in the system ^(8)^. In 2019, PAHO and WHO published a book with a systematic review focused exclusively on improving data use in lowand middle-income countries. This study aimed to identify and publicize what works to improve data use in immunization and why it works^(28)^.

From this perspective, the validation of indicators conducted in our study demonstrated the importance of considering three dimensions (structure, processes, and outcomes) in an integrated manner in the evaluation process of computerized systems in vaccination rooms. Evaluating the structure dimension, the foundation of the system, is crucial to ensure it has the necessary resources to function effectively, as the absence of a solid infrastructure can lead to process failures and compromise desired outcomes. The process dimension evaluation aims to ensure that activities are performed efficiently, safely, and in accordance with best practices, acknowledging that a good structure is ineffective without well-defined processes. The outcome dimension evaluation is essential to determine the system’s success in achieving the proposed objectives and its impact on public health, as the final results are the reason for any vaccination system’s existence. Thus, assuming that each of these dimensions plays a crucial role in the overall evaluation of the computerized system in vaccination rooms, the study results can potentially contribute to the efficiency and effectiveness of vaccination management in everyday health services and, consequently, strengthen PNI actions in the territories.

In recent decades, the availability of vaccines in the global market has grown significantly, leading to frequent updates in the immunization schedule with the introduction of new immunobiologicals. This situation has created the constant need and challenge of monitoring the data on administered doses to evaluate vaccination coverage and its homogeneity across various territories. In this context, the importance of an efficient information system capable of monitoring vaccination services and producing reliable indicators stands out^(7,14)^.

Historically, the development of health information systems in the SUS has resulted from the need for management tools, whether for monitoring or financing health actions and events. However, these initiatives often do not meet the actual needs of services and their professionals, proving inadequate for decision-making processes^(29)^.

In Brazil, the National Vaccination Calendar currently includes 19 vaccines (which protect individuals against more than 20 diseases), available in over 36,000 vaccination rooms in the SUS public network. Daily, the teams in these rooms are responsible for welcoming and assisting users, informing them about available immunobiologicals, checking vaccination cards, administering the immunobiologicals, and recording the administered doses in the information system. Additionally, these professionals are responsible for planning and providing local supplies and immunobiologicals, maintaining the optimal conservation of vaccines, ensuring the proper functioning of equipment, conducting active searches for absentees, and systematically monitoring vaccination coverage^(30,31)^.

In this context, recording data in a computerized system allows for the replacement of large volumes of printed and manual data, creating an expectation of changes and improvements in immunization services. These improvements include faster issuance of reports to evaluate vaccination coverage, identification of absentees, and location of data from lost vaccination cards, among others. With this perspective, municipalities across the country have been encouraged by the PNI to implement computerized vaccination systems in all vaccination rooms, including through financial incentives to states and/or municipalities^(32)^. This initiative aims to improve the quality of immunization data, ensure consistent and timely data entry by all vaccination rooms in Brazilian municipalities, and guarantee the regular and prompt transmission of data to the national database.

Thus, the recording of vaccines administered in Primary Care units began to be done through the Citizen’s Electronic Medical Record (PEC), the Simplified Data Collection (SDC), or in proprietary systems duly integrated with the Health Information System for Primary Care (SISAB). To monitor vaccination coverage, the “reports” modules in SIPNI Web and the SIPNI Tabnet were made available. Data related to the movement of immunobiologicals in vaccination rooms, post-vaccination adverse events, and rapid coverage monitoring remained in SIPNI^(12)^.

In lowand middle-income countries, the low quality of vaccination data is a real issue, and various factors can impact this situation. Besides often insufficient computer training, healthcare professionals in these countries face challenges in data collection, which directly affects the quality of information^(33,34)^.

Due to the socioeconomic heterogeneity among the regions of the country, the challenge for national policies is to ensure uniform implementation of systems and their updates across the entire territory, as well as to expand the capacity for on-site evaluation. In this direction, this study presented a set of valid indicators that can contribute to the evaluation of computerized systems in vaccination rooms, according to PNI recommendations, in any Brazilian municipality with a functioning computerized system in its vaccination rooms.

### Study Limitations

The low response rate of specialists is among the limitations of the Delphi technique, with an expected abstention rate of around 20 to 50% among respondents in each round^(22)^. Despite efforts to maintain the participation of expert judges in both rounds of indicator validation, there was high abstention in the second round, which constitutes a limitation of the study. This abstention can be attributed to the fact that the Delphi method was applied during the COVID-19 pandemic, coinciding with the implementation of COVID-19 vaccination in municipalities across the country. It is known that professionals directly involved in the vaccination campaign during the pandemic experienced a workload overload^(35)^, which may have hindered their continued participation in the research, especially after the first round.

### Contributions to Nursing

Nevertheless, the study made contributions to the field of public health and, particularly, to nursing, as nursing professionals predominantly carry out vaccinations and use evaluation methods in vaccination rooms and in planning the actions to be performed by the immunization service^(31)^.

## FINAL CONSIDERATIONS

The construction of indicators for evaluating the implementation of computerized systems in vaccination rooms followed rigorous methodology to provide a foundation for use by professionals involved in the management, coordination, and supervision of vaccination rooms throughout the country.

The use of the Delphi technique presented the advantage of bringing together a panel of experts who work in different territories, occupying various hierarchical positions, including managers and technical staff with diverse knowledge, perspectives, and professional experiences. Thus, with the validation of the indicators by these experts, it becomes possible for professionals or managers to use these indicators to evaluate the computerized system of their vaccination rooms, in any part of the country, as the indicators adhered to PNI recommendations.
