# Supplementary material for: Study for the validation of evaluation indicators of electronic health records in immunization: Delphi technique
Source: Rev Bras Enferm. 2024 Nov 22;77(5):e20230112. doi: 10.1590/0034-7167-2023-0112 (PMC11653872; doi:10.1590/0034-7167-2023-0112)
Supplement: Translation

# Translation: Estudo de validação de indicadores avaliativos de registros eletrônicos de saúde em imunização: técnica Delfos

**DOI:** 10.1590/0034-7167-2023-0112pt

**Authors:** Cleide Henriqueta Praxedes Fernandes, Maria Aparecida Araújo Figueiredo

**Affiliations:** I Escola de Saúde Pública da Bahia Profº Jorge Novis. Salvador, Bahia, Brasil; II Universidade do Estado da Bahia. Salvador, Bahia, Brasil

**Keywords:** Estudo de Validação, Programa Nacional de Imunizações, Registros Eletrônicos de Saúde, Tecnologia da Informação, Técnica Delfos

## Abstract

**Objetivo:**

Desenvolver e validar indicadores para avaliação de sistema informatizado em salas de vacina.

**Métodos:**

Estudo metodológico. A partir da construção de modelo lógico de gestão da informação produzida em sistema informatizado em salas de vacina, foi elaborada matriz de indicadores de avaliação, cujos conteúdos foram validados por especialistas utilizando o método Delphi. Foram julgados grau de relevância e clareza, tendo como parâmetro: percentual de concordância ≥ 90%; índice de validade de conteúdo > 0,78. A consistência interna foi testada pelo coeficiente de Cronbach de 0,93.

**Resultados:**

Dos 55 indicadores propostos, 48 foram validados e compuseram a matriz final, sendo 13 na dimensão estrutura, 29 na dimensão processo e seis na dimensão resultado.

**Considerações finais:**

O conjunto de indicadores apresenta validade e alta confiabilidade, podendo ser utilizado para avaliar sistema informatizado em salas de vacina em qualquer parte do país, uma vez que respeitou as recomendações do Programa Nacional de Imunizações.

## INTRODUÇÃO

No Brasil, o desenvolvimento tecnológico dos sistemas de informação tem sua trajetória marcada por avanços e retrocessos. Embora se notem esforços no sentido de aprimorar os sistemas existentes para alcançar melhor qualidade do registro, coleta e processamento dos dados, ainda é preciso enfrentar entraves referentes à operacionalização desses sistemas, bem como ao cumprimento do papel das informações produzidas no contexto da saúde ^(1)^. Não obstante, em face das diferentes realidades dos diversos estados brasileiros, a Política Nacional de Informação e Informática em Saúde direcionou as tecnologias de informação e comunicação utilizadas no Sistema Único de Saúde (SUS), favorecendo a padronização dos meios de coleta e tratamento dos dados, bem como a redução da heterogeneidade dos diversos sistemas de informação existentes ^(2)^.

No âmbito do Programa Nacional de Imunizações (PNI), as primeiras ações de sistematização das informações sobre imunização na vigilância epidemiológica iniciaram em 1975, quando o Sistema de Registro de Doses Aplicadas foi implantado ^(3)^. Por muitos anos, os registros permaneceram em papel, acarretando limitações para a disponibilidade dos dados em tempo real, com possibilidade de deterioração da integridade, comprometimento da qualidade das informações, complexidade para recuperação de dados, suscetibilidade a vieses nas informações e obstáculos à pronta pesquisa de dados individuais dos pacientes ^(4)^.

Com o avanço tecnológico, cresceu o interesse global em substituir registros físicos por registros eletrônicos em todos os níveis de atendimento do sistema de saúde, incluindo os serviços de imunização ^(5)^. Assim, acompanhando a tendência global de informatização, o Brasil desenvolveu o Sistema de Informação Nominal do Programa Nacional de Imunizações (SIPNI), cujo objetivo principal é o registro individual dos dados de vacinação de todo brasileiro.

A introdução de um sistema informatizado na área de imunização permitiu a produção de registros oficiais de vacinação de forma ágil, tanto para os serviços quanto para a gestão, gerando informações detalhadas que vão desde a movimentação dos imunobiológicos em cada sala de vacinação, seja na rede pública ou privada, até o acompanhamento do histórico de vacinação de cada cidadão ^(6,7)^. O sistema permite monitorar individualmente o status vacinal, identificar pessoas em atraso vacinal, enviar automaticamente alertas sobre doses vencidas e lembretes para as próximas doses a serem administradas ^(8)^.

No cotidiano, o programa de imunização enfrenta desafios significativos, como a necessidade de identificação da população não vacinada ou subvacinada. Nesse sentido, a implementação de um sistema informatizado em imunização trouxe, como benefício, a possibilidade de identificar essa população, de modo a garantir que todos sejam adequadamente imunizados, favorecendo a manutenção de níveis elevados de coberturas vacinais e a diminuição dos casos de doenças imunopreveníveis ^(9,10)^.

Desse modo, existe uma busca global pela efetivação do registro eletrônico em imunização. Alguns países encontram-se em fase avançada de utilização dos sistemas informatizados em imunização, sendo que alguns são sistemas individualizados e outros atuam como componentes integrados a sistemas informatizados de gestão em saúde ^(11)^.

No Brasil, com o intuito de melhorar a qualidade dos dados de imunização, bem como viabilizar a alimentação do referido sistema de informações em todas as salas de vacina dos municípios brasileiros, garantindo oportunidade da digitação e regularidade da transmissão dos dados para a base nacional, em setembro de 2019, a Portaria nº 2.499 alterou o registro de dados em imunização para apenas um sistema, o e-SUS Atenção Básica (e-SUS AB) ^(12)^. Essa integração, que trouxe importantes mudanças na disponibilização automática de informações sobre o usuário da saúde, também trouxe a vantagem de organizar os dados do cidadão em um mesmo lugar, por meio do prontuário eletrônico, permitindo aos profissionais da Atenção Primária acompanhar o histórico de vacinação dos usuários dos serviços de saúde. Além disso, a integração do SIPNI ao e-SUS AB acrescentou e/ou otimizou algumas funcionalidades com vistas à melhoria dos registros e acompanhamento da situação vacinal dos indivíduos, como, por exemplo, o aprazamento das doses subsequentes, que passou a ser de forma automática ^(13)^.

Todavia, sendo o SIPNI uma inovação tecnológica de gestão das ações de imunização, faz-se necessária a adoção de iniciativas de sensibilização, capacitação, supervisão e avaliação da utilização do recurso em sua totalidade. Há ainda a necessidade do reconhecimento de que o aprimoramento das condutas na operacionalização do sistema apoiará a implementação de ações de vigilância mais efetivas ^(14)^.

Dentro desse dinâmico contexto de implantação e implementação de sistemas de informação, é fundamental ter parâmetros de avaliação. Para Contandriopoulos e colaboradores ^(15)^, durante o desenvolvimento de sistemas e serviços de saúde, é importante a realização de avaliações que busquem fazer o julgamento de valor sobre a operacionalização adequada da intervenção, estabelecendo uma comparação entre as características planejadas e as efetivamente implantadas.

Com essa perspectiva, a principal justificativa para a realização deste estudo se pautou na necessidade de construir e validar indicadores capazes de avaliar sistemas informatizados em salas de vacina, tomando como parâmetro as recomendações do PNI para as boas práticas de execução das ações de vacinação; para o desempenho das salas de vacinação por intermédio do monitoramento das atividades diárias e mensais; bem como para a gestão dos imunobiológicos. A construção e validação desses indicadores permitirá a avaliação de sistemas informatizados de sala de vacina que, por terem utilizado os parâmetros do PNI, poderão ser utilizados em municípios de todo o país.

## OBJETIVO

Desenvolver e validar indicadores para avaliação de sistema informatizado em salas de vacina.

## MÉTODOS

### Aspectos Éticos

Por se tratar de pesquisa na área da saúde, atendeu-se às exigências das Resoluções nº 466/2012 e 510/2016, do Conselho Nacional de Saúde, que dispõe sobre a pesquisa com seres humanos. A pesquisa foi encaminhada para apreciação do Comitê de Ética e Pesquisa da Universidade do Estado da Bahia, sendo aprovada em 27 de maio de 2020.

### Desenho, período e local do estudo

Estudos que buscam a validação e a avaliação de ferramentas, ou métodos de pesquisa, são considerados estudos metodológicos ^(16,17)^. Desse modo, trata-se de uma pesquisa metodológica estruturada para elaboração e validação de conteúdo de indicadores para avaliação do sistema informatizado em salas de vacina. A pesquisa foi desenvolvida no período de dezembro de 2020 a janeiro de 2021, no estado da Bahia, Brasil.

### População e critérios de seleção

Para a seleção de juízes especialistas que iriam validar os indicadores construídos para avaliação de sistema informatizado em salas de vacina, tendo como parâmetro as recomendações do PNI, considerou-se, principalmente, o conhecimento teórico e a experiência prática, elegendo-se como critérios: i) terem formação na área da saúde, ou áreas afins; ii) atuar na gestão do serviço de imunização em qualquer das esferas de governo (municipal, estadual ou federal), com experiência na área, igual ou maior que dois anos. A partir desses critérios, os possíveis especialistas foram escolhidos de forma intencional, contatados via correio eletrônico e/ou mensagens de *Whatsapp*®, por meio de carta-convite encaminhada eletronicamente, com explicações sobre o estudo. Foram excluídos aqueles que não responderam à carta-convite e ao questionário dentro do prazo estabelecido para devolução (15 dias após o primeiro contato).

Para os que aceitaram colaborar, foram encaminhados o termo de consentimento livre e esclarecido (TCLE) e o questionário. O material direcionado a esses juízes foi estruturado em duas partes: a primeira com questões para a caracterização dos participantes e a segunda com o questionário e as instruções de preenchimento.

O grupo de juízes especialistas foi composto por 12 enfermeiros e um técnico de enfermagem, sendo dois mestres, 10 com especialização e um profissional de nível médio. Todas eram do sexo feminino, com maioria autodeclarada de cor parda (69,2%), na faixa etária entre 31 a 40 anos (76,9%), atuando no serviço de imunização, ou no PNI, entre 05 a 15 anos (53,8%). Em relação ao nível de atuação, 84,6% atuava no nível de gestão municipal, sendo cinco no serviço de imunização em unidades de saúde; duas em distritos sanitários e quatro nas coordenações municipais. As demais (duas) eram coordenadores estaduais de imunização.

### Protocolo do estudo

A fase preliminar de construção dos indicadores foi composta por uma revisão integrativa da literatura, acompanhada por levantamento bibliográfico e análise documental sobre as recomendações do PNI para o sistema informatizado nas salas de vacina. A revisão integrativa buscou identificar qual era a abordagem adotada na avaliação dos sistemas informatizados em imunização em diversos países e quais aprendizados podem ser extraídos dos principais resultados e conclusões dessas avaliações para informar melhores práticas e aprimoramento desses sistemas.

Os descritores de busca “Programas de Imunização”, “Avaliação em Saúde”, “Imunização” e “Sistema de Informação em Saúde”, foram combinados em cinco sequências com o emprego do operador booleano “*and*”. Foram incluídas na revisão as produções disponíveis gratuitamente na íntegra em formato digital, nos idiomas inglês, espanhol e português, que abordavam a avaliação do sistema informatizado em imunização em qualquer contexto geográfico, entre janeiro de 2015 a agosto de 2020, e excluídas as produções que não respondiam total ou parcialmente à questão norteadora da revisão ou que tratavam da avaliação de sistemas informatizados em saúde não relacionados com a imunização. Além dos artigos científicos, também foram incluídos documentos não convencionais que compõem a literatura cinzenta (teses, dissertações, anais de simpósios e congresso, manuais e publicações oficiais - notas informativas, portarias e decretos).

O período da coleta ocorreu de abril de 2019 a agosto de 2020, sendo realizada por apenas 01 revisor. Os documentos selecionados foram avaliados quanto à sua relevância para o tema da revisão e os dados foram extraídos e organizados em uma planilha descritiva, incluindo informações como título, autor, ano, tipo de publicação, periódico, local de publicação, objetivos, metodologia, resultados, conclusões e principais achados. Essa análise permitiu uma compreensão mais aprofundada dos estudos avaliativos identificados.

O processo de revisão integrativa envolveu a identificação, triagem, elegibilidade e inclusão de estudos relevantes. Inicialmente, foram identificados 533 registros por meio da pesquisa em banco de dados da Biblioteca Virtual em Saúde e 18 registros adicionais através do *Google Scholar*. Após a remoção de duplicatas, 497 registros foram submetidos à triagem com a leitura dos títulos e resumos, resultando na exclusão de 428 registros. Em seguida, 69 artigos de texto completo foram avaliados para elegibilidade, dos quais 16 foram excluídos com base nos critérios predefinidos.

Ao fim, foram analisados um total de 53 documentos que tratavam sobre o Sistema Informatizado em Imunização sendo 34 artigos, duas teses de doutorado, cinco dissertações de mestrado, três anais de simpósio, um editorial, dois livros, um manual, um Decreto Presidencial, uma Nota Informativa Ministerial e três Portarias Ministeriais. A partir dessa análise documental foram identificados elementos necessários para o funcionamento de sistemas informatizados em salas de vacina. Com esses elementos, criou-se o modelo lógico, base para a construção dos indicadores.

O modelo lógico de um programa ajuda na sua descrição, pois delineia como este será implementado e quais são os resultados esperados ^(18)^. No modelo lógico aqui construído (Figura 1) foram observadas as dimensões donabedianas de avaliação ^(19)^, que considera: a estrutura como dimensão relacionada aos recursos humanos, materiais e organizacionais necessários para o funcionamento e implantação do sistema; a dimensão processo sendo aquela que busca discriminar as atividades desenvolvidas no funcionamento; e a dimensão resultados descrevendo aquilo que se pretende alcançar. Em seguida foram estabelecidos dois componentes principais: i) alimentação do sistema informatizado; ii) gestão da informação. Os subcomponentes da alimentação do sistema informatizado foram três: i) triagem, ii) registro do vacinado e iii) movimento de imunobiológicos. Para gestão da informação os subcomponentes foram: i) monitoramento das atividades diárias e ii) monitoramento das atividades mensais. Com base neste modelo lógico definiu-se a matriz de indicadores.

**Figura 1:** Modelo Lógico

Indicadores são recursos metodológicos, quantitativos ou qualitativos, que permitem a análise de variáveis, ou conjunto de variáveis, que constituem o objeto observado, importantes para o enfrentamento de um problema, sendo necessário, entretanto, considerar na sua definição sua relevância e utilidade ^(20)^. Com essa perspectiva, para cada dimensão (estrutura; processo; resultado), e suas respectivas subdimensões, foi criado pelo menos um indicador, com seus respectivos parâmetros de avaliação e critérios de pontuação, constituindo, assim, a denominada “matriz de indicadores”. Essa matriz foi formada por 55 indicadores, sendo 14 propostos para a dimensão “estrutura”, 35 para a dimensão “processo” e seis para a dimensão “resultados”.

Para a validação do conteúdo e obtenção do consenso acerca da matriz de indicadores, foi utilizado o método Delphi - definido como um processo de comunicação estruturado que busca obter consenso de opiniões de um grupo de especialistas sobre determinado tema, por meio de questionários intercalados com o *feedback* de opinião controlada. O método pressupõe que a opinião do coletivo de especialistas, quando devidamente organizada, sobrepõe ao julgamento individual ^(21,22)^. Aqui buscou-se seguir o e-Delphi Modificado que consiste em processo semelhante ao Delphi clássico, administrado por e-mail, ou online, com pesquisas web ^(23)^. Assim, a matriz foi enviada aos especialistas por meio de planilha eletrônica *Google Forms*®.

No início da aplicação do método Delphi é importante a estruturação da nota de instrução para os participantes, com esclarecimentos sobre a execução da investigação, a conformação dos questionários, os critérios e a forma da avaliação ^(23)^, entendendo que o consenso foi alcançado no momento em que as opiniões dos juízes convergem ^(24)^.

Assim, foi solicitado aos juízes que avaliassem cada um dos indicadores segundo a sua relevância e clareza, com a seguinte escala: i) relevância - compreendida como o grau de importância (alto para os indicadores imprescindíveis, sem os quais não é possível oferecer o serviço previsto por contemplarem requisitos fundamentais ao seu funcionamento; médio para os indicadores necessários, que devem estar presentes e que apontam para adoção de boas práticas na organização e funcionamento do serviço, porém não são imprescindíveis; baixo para os indicadores que podem estar presentes na organização e no funcionamento do serviço, mas que necessitam de grande revisão para ser relevante; e nulo para os indicadores não relevantes); ii) clareza avaliando se o indicador está redigido com clareza, simplicidade, facilidade e naturalidade, sendo sim, caso considere a redação da questão clara, e não ao julgá-lo não claro.

Durante a primeira rodada, dos 26 profissionais contactados, 14 responderam à carta convite. Destes, um não respondeu ao questionário perfazendo para a primeira rodada um grupo composto por 13 profissionais juízes, resultando assim numa abstenção de 7,14%. Para a segunda rodada houve uma abstenção de 53,8 %, apenas seis especialistas dentre os 13 foram respondentes.

### Análise dos resultados e estatística

Os dados foram armazenados em planilha do Excel®. Para a validação de conteúdo de um instrumento existem diferentes métodos para quantificar o grau de concordância entre os juízes especialistas ^(25)^. Para este estudo foram utilizados o percentual de concordância entre os juízes e o Índice de Validade de Conteúdo (IVC). Assim, seguindo o recomendado por Polit e Beck, os indicadores com percentual de concordância inferior a 90% (para o item clareza) ou com IVC menor que 0,78 (para o item relevância), foram reformulados ou excluídos do instrumento ^(16)^. As seguintes fórmulas foram utilizadas:


$$\text{Porcentagem de concordância}=\frac{\text{Nº de participantes que concordam (sim) quanto à clareza X 100}}{\text{Nº total de participantes}}$$

$$\text{IVC}=\frac{\text{Nº de respostas que consideraram a relevância alta ou média}}{\text{Nº total de repostas (relevância alta, média, baixa ou nula)}}$$

Para estimar a medida de consistência interna das perguntas e respostas, foi calculado o coeficiente de Cronbach no pacote estatístico de domínio livre denominado R, cujos valores desejáveis variam ente 0,70 e 1,0 ^(26)^.

## RESULTADOS

Dos 26 profissionais contactados, 14 responderam à carta-convite. Destes, um não respondeu ao questionário, perfazendo, para a primeira rodada, um grupo de 13 especialistas, com 7,1% de abstenção. Para a segunda rodada, houve uma abstenção de 53,8%, pois, dos 13 participantes da primeira rodada, apenas seis especialistas responderam.

Na primeira rodada, dos 55 indicadores, 80% obtiveram percentual de concordância e IVC acima dos valores preestabelecidos, sendo assim considerados como tendo conteúdo válido. Para a dimensão estrutura, que tratou dos recursos estruturais, humanos, materiais e organizacionais, dos 14 indicadores existentes, 12 foram validados na primeira rodada e dois não obtiveram consenso quanto à sua clareza e/ou relevância, a saber: realizar ou solicitar a inclusão de novos profissionais no sistema informatizado; inexistência de profissional cadastrado no sistema informatizado que já não compõe o quadro funcional. Os não validados foram reformulados e reapresentados aos juízes na segunda rodada, sendo um excluído nesta etapa (Quadro 1).

**Quadro 1:** Indicadores do sistema informatizado em sala de vacina referentes à dimensão estrutura não validados na primeira rodada do método Delphi e situação após a segunda rodada

Indicador apresentado na 1ª rodada	Resultado 1ª rodada Delphi		Indicador reformulado e apresentado na 2ª rodada	Resultado final
	IVC	% Concordância		
Inexistência de profissional cadastrado no SI que já não compõe o quadro funcional.	0,77	84,6%	Profissionais que já não compõem o quadro funcional da sala de vacina são excluídos do SI.	Indicador excluído.
Realizar ou solicitar de inclusão de novos profissionais no SI	0,92	84,6%	Todos os profissionais que atuam na sala de vacina são cadastrados no SI.	Indicador validado. (ICV =1 e % Concordância = 100%)

*Notas: SI - Sistema de Informação; IVC - Índice de Validade de Conteúdo.*

Ao analisarmos os 35 indicadores referentes à dimensão processo, que tratou das atividades realizadas na operacionalização do sistema informatizado, 29 foram validados na primeira rodada, um não alcançou IVC > 0,78 e cinco tiveram concordância inferior a 90% (Figura 2), migrando, então, para a segunda rodada. Destes, cinco foram validados e um excluído nesta etapa (Quadro 2).

**Quadro 2:** Indicadores do sistema informatizado em sala de vacina referentes à dimensão processo não validados na primeira rodada do método Delphi e situação após a segunda rodada

Indicador apresentado na 1ª rodada	Resultado 1ª rodada Delphi		Indicador reformulado e apresentado na 2ª rodada	Resultado final
	IVC	Concordância		
Inclusão de informações não obrigatórias no cadastro do usuário.	0,77	92,3%	Reapresentado na íntegra	Indicador excluído.
Registro no SI da via de administração do imunobiológico.	0,92	84,6%	Inclui no SI dados sobre a via de administração da vacina.	Indicador validado. (ICV =1 e % Concordância = 100%)
Consulta no SI quantidade de doses perdidas por quebra de frascos.	0,85	84,6%	Visualiza no SI o registro de doses perdidas por quebra de frascos.	Indicador validado. (ICV =0,83 e % Concordância = 100%)
Consulta no SI quantidade de doses perdidas por falha no transporte.	0,85	84,6%	Visualiza no SI o registro de doses perdidas por falha no transporte.	Indicador validado. (ICV =0,83 e % Concordância = 100%)
Consulta no SI quantidade de doses perdidas por perdas técnicas ou outros motivos.	0,85	84,6%	Visualiza no SI o registro de doses perdidas por perdas técnicas ou outros motivos.	Indicador validado. (ICV =0,83 e % Concordância = 100%)
Verifica no sistema informatizado relação de aprazamentos e faltosos.	0,85	84,6%	A relação de pacientes faltosos e de aprazamentos das doses subsequentes são visualizadas no SI.	Indicador validado. (ICV =1 e % Concordância = 100%)

*Notas: SI - Sistema de Informação; IVC - Índice de Validade de Conteúdo*

**Figura 2:** Concordância entre especialistas para os indicadores de implantação do sistema informatizado em sala de vacina referentes à dimensão PROCESSO na primeira rodada do método Delphi, segundo IVC e % de Concordância

Dos seis indicadores que compunham a dimensão resultado, que trata do funcionamento dos relatórios gerenciais que espelham os objetivos do sistema informatizado, três indicadores foram validados na primeira rodada, a saber: confecciona o boletim mensal apenas com dados disponíveis no sistema informatizado; revisa arquivos de informações individuais do vacinado registrados no sistema informatizado de indivíduos acompanhados pela unidade; emite relatório de vacinados por vacina. Os outros três foram reformulados, sendo validados na segunda rodada (Quadro 3).

**Quadro 3:** Indicadores do sistema informatizado em sala de vacina referentes à dimensão resultado não validados na primeira rodada do método Delphi e situação após a segunda rodada

Indicador apresentado na 1ª rodada	Resultado 1ª rodada Delphi	Indicador reformulado e apresentado na 2ª rodada	Resultado final
Confecciona o boletim mensal apenas com dados disponíveis no SI.	ICV = 0,85 Concordância = 84,6%	Boletim Mensal é confeccionado exclusivamente com dados do SI.	Indicador validado. ICV = 0,83 e % Concordância = 100%
Revisa arquivos de informações individuais do vacinado registrados no SI de indivíduos acompanhados pela unidade.	ICV = 0,85 Concordância = 84,6%	Revisa arquivos de informações dos vacinados registrados no SI acompanhados pela unidade.	Indicador validado. ICV =1 e % Concordância = 100%
Emite relatório de vacinados por vacina.	ICV = 0,85 Concordância = 84,6%	Visualiza no SI a relação de vacinados por cada imunobiológico.	Indicador validado. ICV =1 e % Concordância = 100%

*Notas: SI - Sistema de Informação; IVC - Índice de Validade de Conteúdo*

A matriz final foi formada por 48 indicadores, sendo 13 para a dimensão ESTRUTURA, 29 para a dimensão PROCESSO e seis para a dimensão RESULTADO. O coeficiente alfa de Cronbach (dados não demonstrados) atingiu 0,93 (IC 95%), revelando um valor significativamente elevado. Esta constatação sugere uma consistência interna robusta para o questionário, indicando uma excelente confiabilidade interna no que diz respeito à clareza e relevância dos indicadores.

## DISCUSSÃO

No âmbito das publicações sobre avaliações do sistema informatizado em salas de vacina, um estudo trouxe a experiência da Organização Mundial da Saúde (OMS), que em 2001 desenvolveu uma ferramenta de autoavaliação adaptando a metodologia de Auditoria da Qualidade dos Dados para uso em programas nacionais, com a finalidade de apoiar os países na melhoria da coleta e uso de dados para diagnosticar problemas em todas as esferas de gestão (federal, estadual, municipal ou local). Essa metodologia foi aprimorada em 2014, em parceria com o Centro de Controle e Prevenção de Doenças dos Estados Unidos (CDC) ^(8,27)^. Nessa direção, outro estudo relatou a experiência da Organização Pan-Americana da Saúde (OPAS) no desenvolvimento de uma metodologia para avaliar os registros eletrônicos de vacinação em países de baixa e média renda na América Latina e no Caribe, partindo das discussões sobre o que seria considerado como “ideal” para um sistema informatizado em imunização, considerando as seguintes dimensões avaliadas: i) propósito do sistema; ii) contexto legal e normatização; iii) funcionamento do software; iv) manutenção; v) sustentabilidade; vi) recursos humanos; e vii) módulos incluídos no sistema ^(8)^. Em 2019, a OPAS e a OMS publicaram um livro com uma revisão sistemática tratando exclusivamente da melhoria do uso de dados em países de baixa e média renda. Tal estudo buscou identificar e publicizar o que funciona para melhorar o uso dos dados em imunização e por que funciona ^(28)^.

Com essa perspectiva, a validação dos indicadores realizada em nosso estudo demonstrou a importância de considerar três dimensões (estrutura, processos e resultados), de maneira integrada, no processo de avaliação de sistema informatizado de salas de vacina. A avaliação da dimensão estrutura, base do sistema, é fundamental para garantir que este tenha os recursos necessários para funcionar eficazmente, uma vez que a ausência de uma infraestrutura sólida pode ocasionar falhas nos processos e comprometer os resultados desejados. Já a avaliação da dimensão processo visa garantir que as atividades sejam executadas de maneira eficiente, segura e de acordo com as melhores práticas, considerando ainda que uma boa estrutura é ineficaz sem processos bem definidos. Por sua vez, a avaliação da dimensão resultados é crucial para determinar o sucesso do sistema em alcançar os objetivos propostos e seu impacto na saúde da população, visto que os resultados finais são a razão de ser de qualquer sistema de vacinação. Assim, pressupondo que cada uma dessas dimensões desempenha um papel crucial na avaliação global do sistema informatizado em sala de vacina, os resultados do estudo podem contribuir potencialmente para a eficiência e eficácia na gestão da vacinação no cotidiano dos serviços de saúde e, consequentemente, fortalecer as ações do PNI nos territórios.

Nas últimas décadas, a disponibilidade de vacinas no mercado mundial cresceu significativamente, de modo que o cronograma de imunização vem sendo atualizado frequentemente com a introdução de novos imunobiológicos. Tal situação gerou a necessidade e o desafio constantes de monitorar os dados de doses aplicadas, a fim de avaliar as coberturas vacinais e sua homogeneidade nos diversos territórios. Nesse contexto, destaca-se a importância de um sistema de informação eficiente, capaz de monitorar o serviço de vacinação e produzir indicadores fidedignos ^(7,14)^.

Historicamente, o desenvolvimento dos sistemas de informação em saúde no SUS tem sido resultado da necessidade de ferramentas de gestão, seja no âmbito do monitoramento, seja no âmbito do financiamento de ações e eventos de saúde. Contudo, quase sempre, essas iniciativas não atendem às verdadeiras necessidades dos serviços e de seus profissionais, apresentando-se incipientes para os processos de tomada de decisões ^(29)^.

No Brasil, atualmente, existem estabelecidas na rotina do Calendário Nacional de Vacinação 19 vacinas (que protegem os indivíduos para mais de 20 doenças), disponíveis nas mais de 36 mil salas de vacina da rede pública do SUS. Diariamente, as equipes de trabalho dessas salas se responsabilizam por acolher e assistir o usuário, orientando-os sobre os imunobiológicos disponíveis, conferindo as carteiras de vacinação, aplicando o imunobiológico, indicando e registrando as doses aplicadas no sistema de informação. Além disso, esses profissionais são também responsáveis por planejar e prover as necessidades locais de insumos e imunobiológicos, manter a conservação ideal das vacinas, zelar pelos equipamentos e suas condições de funcionamento, realizar a busca ativa de faltosos e acompanhar sistematicamente as coberturas vacinais ^(30,31)^.

Nesse contexto, o registro de dados em sistema informatizado possibilita a substituição de grande volume de impressos e dados manuais, gerando uma expectativa de mudanças e melhoria no serviço de imunização, tais como agilidade na emissão de relatórios que permitam a avaliação das coberturas vacinais, a identificação de faltosos e a localização de dados de cartões de vacinação perdidos, dentre outros. Com essa perspectiva, os municípios de todo o país vêm sendo estimulados pelo PNI a implantar sistemas informatizados de vacinação em todas as salas de vacina, inclusive através de incentivos financeiros aos estados e/ou municípios ^(32)^. Tal iniciativa busca melhorar a qualidade dos dados de imunização, proporcionar a manutenção da alimentação por todas as salas de vacina dos municípios brasileiros, garantindo a regularidade e oportunidade da digitação, bem como a transmissão dos dados para a base nacional.

Assim, o registro das vacinas administradas nas unidades de Atenção Primária passou a ser realizado por meio do Prontuário Eletrônico do Cidadão (PEC), da Coleta de Dados Simplificada (CDS) ou em sistemas próprios devidamente integrados ao Sistema de Informação em Saúde para a Atenção Básica (SISAB). Para o acompanhamento da cobertura vacinal, foram disponibilizados os módulos “relatórios” no SIPNI Web e o Tabnet do SIPNI. Os dados referentes à movimentação de imunobiológicos nas salas de vacina, aos eventos adversos pós-vacinação e aos monitoramentos rápidos de cobertura permaneceram no SIPNI ^(12)^.

Em países de baixa e média renda, a baixa qualidade de dados de vacinação é um problema real e uma série de fatores pode impactar essa questão. Além das capacitações em informática muitas vezes incipientes, os profissionais de saúde desses países enfrentam desafios para coletar dados, o que implica diretamente na qualidade da informação ^(33,34)^.

Devido à heterogeneidade socioeconômica entre as regiões do país, o desafio para as políticas nacionais está em garantir uma homogeneidade na implantação dos sistemas e suas atualizações em todo o território, bem como ampliar a capacidade de avaliação in loco. Nessa direção, este estudo trouxe um conjunto de indicadores considerados válidos que poderão contribuir para a avaliação de sistemas informatizados em salas de vacina, de acordo com as recomendações do PNI, em qualquer município do Brasil que tenha o sistema informatizado em funcionamento nas salas de vacina.

### Limitações do estudo

A baixa taxa de resposta dos especialistas está entre as limitações da técnica Delphi, sendo esperado que ocorra uma abstenção em torno de 20 a 50% entre os respondentes de cada rodada^(22)^. Mesmo com os esforços empreendidos para garantir a manutenção dos juízes especialistas nas duas rodadas de validação dos indicadores, houve alta abstenção na segunda rodada, o que se configura como uma limitação do estudo. Tal abstenção pode ser atribuída ao fato de a aplicação do método Delphi ter ocorrido durante o período da pandemia de COVID-19, coincidindo com a implantação da vacinação contra COVID-19 nos municípios do país. É sabido que profissionais envolvidos diretamente na campanha de vacinação durante a pandemia tiveram sobrecarga de trabalho^(35)^, o que pode ter dificultado sua permanência na pesquisa, principalmente após a primeira rodada.

### Contribuições para a Enfermagem

Não obstante, o estudo trouxe contribuições para a área da saúde pública e, particularmente, para a enfermagem, uma vez que o profissional de enfermagem é a categoria que predominantemente atua na vacinação e, por isso, utiliza métodos avaliativos das salas de vacina e no planejamento das ações a serem executadas pelo serviço de imunizações ^(31)^.

## CONSIDERAÇÕES FINAIS

A presente construção de indicadores para avaliação da implantação do sistema informatizado em salas de vacina seguiu rigor metodológico, a fim de permitir embasamento para seu uso por profissionais que atuam na gestão, coordenação e supervisão de salas de vacinação de todo o país.

A utilização da técnica Delphi apresentou a vantagem de reunir, no painel de especialistas, pessoas que atuam em diferentes territórios, ocupando cargos hierárquicos distintos, gestores e técnicos da assistência, com saberes, olhares e vivências profissionais diversos. Assim, com a validação dos indicadores por esses especialistas, fica possível sua utilização por profissionais ou gestores que queiram avaliar o sistema informatizado de suas salas de vacina, em qualquer parte do país, uma vez que os indicadores respeitaram as recomendações do PNI.
